# Recent Advances in Cancer Immunotherapy with a Focus on FDA-Approved Vaccines and Neoantigen-Based Vaccines

**DOI:** 10.3390/vaccines11111633

**Published:** 2023-10-25

**Authors:** Anna Hargrave, Abu Salim Mustafa, Asma Hanif, Javed H. Tunio, Shumaila Nida M. Hanif

**Affiliations:** 1 Department of Biomedical Sciences, University of Pikeville, Pikeville, KY 41501, USA; annahargrave@upike.edu; 2 Department of Microbiology, Kuwait University, Kuwait City 12037, Kuwait; abu.mustafa@ku.edu.kw; 3 Department of Restorative Sciences, Kuwait University, Kuwait City 12037, Kuwait; asma.h@hsc.edu.kw; 4 Carver College of Medicine, University of Iowa, Iowa City, IA 52242, USA; javed-tunio@uiowa.edu

**Keywords:** cancer, immunotherapy, recent advances

## Abstract

Cancer immunotherapies refer to the concept of retraining the immune system to target malignant cells. Multiple immunotherapeutic options exist including immune modulating antibodies, immune stimulating cytokines, chimeric antigen receptor T cell therapy, and vaccines. Overall, this field has advanced rapidly as knowledge of the tumor microenvironment, immunological pathways, and biotechnology expands. Specifically, advancements in neoantigen identification, characterization, and formulation into a vaccine show promise. This review is focused on previously United States Food and Drug Administration-approved cancer therapeutic vaccines and neoantigen-based vaccine developments along with the associated relevant clinical trials.

## 1. Introduction

Cancer cells arise from normal cells with uncontrollable growth and unregulated cell cycles. Since these cells are of the same lineage as normal cells, the immune system is unable to recognize the malignancy. The tumor cells produce new proteins, mutate, and evade the immune defense mechanisms, leading to “immunoediting”. Immunoediting refers to the cancerous cells’ ability to selectively produce tumor molecules that will be undetected by the immune response. Besides immunoediting, the tumor cells deliver substances to block the immune cells in the surrounding environment and trigger inflammation with the release of immunosuppressive cytokines [1]. The intricate microenvironment plays a key role in preventing the efficacy of tumor-infiltrating lymphocytes from eliminating the cancerous cells [2].

The purpose of cancer immunotherapy is to retrain the T cells of immune system to overcome the tumor microenvironment, attack, and eliminate cancer [2,3]. Many treatment options fall in this category of immunotherapy: immune modulating antibodies, immune stimulating cytokines, chimeric antigen receptor (CAR) T cell therapy, and immunization strategies. Often, immunotherapy must be used in conjunction with surgery, chemotherapy, and/or radiation because alone it is not curative.

To briefly describe the origin and major advancements of cancer immunotherapy, it began with William Coley’s application of an inactivated bacterial toxin in osteosarcoma. Coley published results in 1893 [4] showing some tumor regression [2] and encouraged further research to prove that vaccines can induce an immune response resulting in tumor clearance. Then, in the early 1900s, Paul Ehlrich’s work regarding the immune surveillance hypothesis laid the foundation for modern immunotherapy [2]. This includes immune stimulating cytokines, immune modulating antibodies, and chimeric T cell therapy, and these all fall under the passive immunotherapy category. [1,2]. The application of immune modulating antibodies has only become an important therapy over the past decade [5].

The difference between passive and active immunotherapy is of fundamental importance. Passive immunotherapy is the concept of “passive acceptance by an organism of antibodies, cytokines, or transformed cells that directly act on the tumor” [1]. Active immunotherapy means that the malignant cells would be directly killed by the immune system, as theorized with a tumor vaccine. Though the advent of these passive therapies have revolutionized medicine, there are still significant challenges with their application and efficacy, particularly with solid tumors [1]. The most important passive immunotherapies in use today are the checkpoint inhibitors as discussed in detail in the following paragraphs.

Immune modulating antibodies or checkpoint inhibitors most commonly target the cytotoxic T-lymphocyte associated protein 4 (CTLA-4) or PD-1/PD-L1 ligand as the cancer cells display these ligands on their cell surface. Without a checkpoint inhibitor, the PD-1/PD-L1 or CTL-4 from the tumor can interact with the T lymphocyte and block the T lymphocyte’s killing ability. Nivolumab, pembrolizumab, atezolizumab, durvalumab, and cemiplimab are checkpoint inhibitors that neutralize the PD-1/PD-L1 pathway and allow the T lymphocyte to resume normal action. Most cancer cells increase PD-L1 expression to evade immune surveillance and exhaust the T lymphocytes. This means that this therapy is more often used, with nivolumab and pembrolizumab the most common in the clinical trials discussed later in this paper [2,5]. Ipilimumab does the same but via the CTLA-4 pathway [6]. The overexpression of CTLA-4 correlates to a worse patient prognosis in breast, thymus, esophageal, and nasopharyngeal cancer. Only a subset of cancers can benefit from anti-CTLA-4 therapy. More research is needed to understand the mechanism behind why [5]. Also, there is research into several other alternative immune modulating targets like TIGIT, LAG-3, or stimulatory immune checkpoints. Small molecular inhibitors and antibodies with these targets are being tested as monotherapy or combined drug therapy in multiple phase 1 and 2 clinical trials, as reviewed by Cooper et al. [7].

The limitation of both types of checkpoint inhibitors is significant. If the tumor has low immune reactivity with no preexisting tumor-specific T cell responses, there is little benefit of using a checkpoint inhibitor as the immune system is unable to terminate the tumor growth [8]. This correlated to a significant number of cancer patients who failed to respond to checkpoint inhibitors (or developed resistance) [5]. Two types of resistance occur: acquired resistance where the cancer progresses on therapy after an initial clinical benefit of 6 months or greater and primary resistance where the tumor progresses after at least 6 weeks but not more than 6 months on therapy. Either resistance type has multiple factors like a clinical phenotype of progression, primary cell of the tumor microenvironment, germline and tumor genetics, prior treatment history, clinical and demographic characteristics [7] It is believed that ultimately the lack of response from some patients occurs due to the heterogenous tumor microenvironment [5].

Given the challenges of checkpoint inhibitors and the possibility of individualized therapy, there is a strong interest into the design of an active therapy or a cancer therapeutic vaccine. This would be classified as an active therapy [1] and ideally would have the following characteristics: react with tumor specific neoantigens, utilize a highly immunogenic vaccine platform, generate a line of expanded and primed T cells, be combined with checkpoint inhibitor therapy, and produce a long-term memory response [8]. The rationale behind combining with checkpoint inhibitor therapy is that the T lymphocytes induced by the vaccine can be blocked through interactions between the tumor cells ligands and inhibitor receptors. Dual therapy with immunization and checkpoint inhibitors would result in a synergistic effect and more effective tumor cell death [9].

Within this classification of active immunotherapy, there are two fundamentally different types of cancer vaccines: therapeutic and prophylactic. Prophylactic vaccines are considered a classic vaccine because they immunize against an oncogenic virus, like Hepatitis B and human papillomavirus. These viruses are well known to be linked to hepatocellular carcinoma and cervical cancer [10]. There is also research into generating prophylactic vaccines for hepatitis C, Epstein–Barr virus, and Kaposi’s sarcoma-associated herpesvirus [11].

Therapeutic vaccines are employed as a treatment strategy for underlying malignancy [12] and are designed to induce host T cells to respond to cancer antigens while interrupting the tolerance acquired by the tumor cells [13]. This targeted immune response against the tumor would spare healthy cells and prevent tumor recurrence through long-term immune cell memory. This type of vaccination may be used as an alternative or an adjunct to traditional treatment [14]. Currently, only three therapeutic anticancer vaccines have been approved by the FDA and are discussed in detail in Section 2 to provide more context of the limited scope and challenges with these immunizations.

Within the category of therapeutic cancer vaccines, there are several subclasses including tumor cell lysates, dendritic cells, nucleic acids, oncolytic viruses, and neoantigens. This review is focused on neoantigen-based immunizations.

To design a vaccine against a tumor, the identification and selection of effective antigens is the first step. Antigens in tumors can be divided into two classes, tumor-associated antigens and neoantigens, as discussed in the following paragraph. Tumor-associated antigens (TAAs) are expressed by both tumor cells and healthy cells [12]. Some examples of TAAs include cancer-testis antigens, HER-2/neu, human epidermal growth factor receptor (EGFR), mucin 1 (MUC1) oncofetal antigens (carcinoembryonic antigen, alpha fetoprotein), and mutated oncoproteins (p53, ras) [12,14].

Neoantigens are not expressed in any other cell other than cancer cells and arise from random genetic mutation or abnormal gene expression. Given that no other cell expresses neoantigens, the immune system can recognize them as non-self and will not induce tolerance [14]. Therapies targeting neoantigens also would not cause any “off-target” adverse effects given the specificity of the expressed location [15,16].

In general, neoantigens can be categorized as shared or personalized. Shared neoantigens are present in select tumor types and could be used in theory to treat those types of tumors. The application of this would be challenging given the antigenic differences across patients and tumors. Personalized neoantigens are present in individual patients and if a vaccine with these personalized neoantigens is generated, it would induce a more specific response [17].

Regardless of whether shared or personalized neoantigens are desired, the first step is neoantigen identification. The most common approach is to utilize next-generation sequencing on a tumor sample and compare the DNA sequence to a normal sample. This poses difficulties because the mutations identified may be noncoding or nonsense mutations. Researchers learned that whole-exome sequencing, where only the protein-encoding part of the genome is sequenced, is a more efficient and feasible method to identify neoantigens, and this method is widely employed [17].

Though the whole-exome sequencing recognizes personalized mutations, there are still steps to predict whether it would be expressed as a protein and able to interact with an MHC molecule during antigen presentation [17]. Several computer simulations, algorithms, and tools have been developed for this purpose. There is growing interest to understand which system is the most accurate and effective at predicting a neoantigen. The preliminary algorithms predicting neoantigens were based on peptide binding affinity data, but this did not predict if the antigen would be displayed on the cell surface to interact with MHC I. To solve this problem, researchers utilized mass spectrometry to analyze peptides interacting with MHC molecules, and this has been used to train prediction algorithms to improve accuracy [16]. Bulik-Sullivan et al. created a computational model, EDGE, from mass spectrometry data of 74 tumor samples. This predicts neoantigens as a deep learning model of HLA peptide presentation. Additionally, it can recognize neoantigen-specific T cells from a limited volume of a patient’s blood sample [18].

The focus of neoantigen-based vaccines in clinical trials has been on neoantigens that theoretically interact with MHC I, with a single trial incorporating MHC II binding neoantigens. Further research is necessary to design an effective vaccine that will induce a tumor-specific CD4+ T cell response [16,19].

The following review discusses FDA-approved therapeutic vaccines, the challenges associated with neoantigen-based therapy (biological complexity, sample collection and associated problems, production cost and time, and adjuvant), a comparison of peptide vs. mRNA vaccines, a summary of the clinical trials of neoantigen-based immunization, the status of a subset of the peptide vs. mRNA vaccines in clinical trials, and future directions.

## 2. FDA-Approved Therapeutic Vaccines

It is important to note that the FDA approved three therapeutic vaccines: Bacillus Calmette-Guerin (BCG) in the 1970s [2,20], Sipuleucel-T in 2010 [2,13], and talimogene laherparepvec in 2015 [12].

BCG is considered the “gold standard treatment” for the treatment of high-risk non-muscle-invasive bladder cancer. However, there are several problems associated with this vaccine because of significant supply problems [21], a poor response to treatment in some patients, and a lack of understanding behind the mechanism(s) of action [20].

Though BCG has been used clinically since 1976, there is a severe shortage currently and the vaccine is incredibly challenging to manufacture due to slow growth. Additionally, within the production line, there are several sub-strains in existence, leading researchers to question which sub-strain is the most superior at treating this type of bladder cancer and preventing recurrence, but no conclusion has been reached [21]. When utilized as a therapy, one dose is equivalent to over four thousand BCG vaccinations and may be administered for one year in certain patient populations. The scarcity may be solved by research into the modification of the therapy schedule, potentially lowering the dosage of BCG and possibly altering the method of drug delivery, but for the time being, BCG production must continue [21].

Even though BCG remains the standard of care, there is a relatively high rate of adverse events and a subset of patients do not respond to this therapy. One clinical trial reported an overall rate of adverse events as high as 70%. Further research is ongoing to investigate alternative treatments for the subset of the BCG-unresponsive group of patients [21].

Though BCG has been employed as a component of therapy for non-muscle-invasive bladder cancer for nearly four decades, the mechanism behind its clinical use remains unclear. It has been hypothesized that the bladder cancer cells internalize the BCG, signal the immune system with cytokines and chemokines, and present BCG and/or cancer antigens to the immune cells. For this to be an effective therapy, the following components are crucial: intact immune system, live BCG, and proximity between BCG and bladder cancer cells. Several immune cells are implicated, including CD4+ and CD8+ lymphocytes, natural killer cells, and granulocytes [20]. The theories of the antitumor mechanism of BCG are explored in detail in “The mechanism of action of BCG therapy for bladder cancer–a current perspective” by Gil Redelman-Sidi [20]. Further investigation into the mechanism may help produce more effective therapeutic treatments.

The remaining two FDA-approved therapeutic cancer vaccines, sipuleucel-T and talimogene laherparepvec, also have similar challenges and unclear mechanisms, as discussed below.

In 2010, sipuleucel-T, a dendritic cell vaccine for metastatic castration-resistant prostate cancer, was approved by the United States FDA [2,22]. Sipuleucel-T is composed of peripheral blood mononuclear cells and antigen-presenting cells that were stimulated by PA2024 ex vivo. PA2024 is a recombinant protein with prostate-specific antigen and prostatic acid phosphatase [17]. Though it has been approved, it remains an expensive and complex treatment [15,22] and it has not been adopted as a mainstay treatment by oncologists and clinical investigators [13]. It modestly improved 36-month survival to 31.7%, compared to 23% in the placebo group [17], and did not significantly decrease tumor volume in randomized clinical trials [13].

Talimogene laherparepvec or T-VEC (tradename IMLYGIC) is the first FDA-approved oncolytic viral drug [1], which received approval in 2015 for advanced melanoma. It is composed of a unique genetically modified live-attenuated herpesvirus with the GM-CSF gene. In patients with recurrent melanoma, the vaccine is injected into unresectable cutaneous, subcutaneous, or nodular lesions and induces local and systemic effects. At the local level, the tumor cells are killed through viral replication and lysis because of the GM-CSF produced and introduced to the dendritic cells and other APCs [2,12]. At the systemic level, the dendritic cells carrying tumor antigens migrate to lymph nodes but overall induce a weaker immune response as compared to the local-level response [12].

## 3. Neoantigens as Vaccine Targets

Neoantigens are newly formed antigens by tumor cells which are missing in the normal cells. These antigens are produced by tumor cells because of various tumor-specific alterations, e.g., genomic mutation, dysregulated RNA splicing, disordered post-translational modification, and integrated viral open reading frames, etc. Since neoantigens are absent in normal cells, they are recognized by immune cells as non-self and induce immune responses. The prediction of tumor-specific neoantigens is possible due to the fast and cost-effective detection of tumor-specific mutations by using next-generation nucleic acid sequencing technologies and the application of bioinformatic tools. The identified tumor-specific neoantigens are considered appropriate targets for personalized cancer immunotherapies [23]. However, the development of such immunotherapies will require a thorough understanding of the mechanisms responsible for neoantigen-induced anti-tumor immunity so that successful neoantigen-based immunotherapies are developed and applied for the treatment of cancer patients. 

In general, various genetic alterations/mutations in the growing cancer cells lead to the production of neoantigens. These tumor-specific antigens/peptides can combine with histocompatibility complex molecules of tumor cells for presentation to T cells to induce an anti-cancer immune response in cancer patients. An interesting characteristic of neoantigen-specific T cells is that they can bypass negative selection effects in the thymus due to the highly antigenic neoantigens produced due to somatic tumor mutations. Increasing the quantity of neoantigen-specific T cells due to this ability to avoid T cell central tolerance makes it possible to enhance tumor-specific immune responses [24]. Furthermore, the capacity of immunotherapy-enhanced neoantigen-specific T cell responses are long-lasting due to the induction of immunological memory that offers hope for long-term protection against tumor recurrence [16].

## 4. Challenges of Neoantigens

Though neoantigen-based therapeutic strategies are certainly beneficial, there are still several challenges associated with this technology. These challenges are the inherent biological complexity of the neoantigens due to the mutations from genetic instability during tumorigenesis, issues with sample collection, identification process (as previously discussed in the introduction), delivery platform, production cost and time, and most appropriate adjuvant(s) [3,17,19]. Additionally, the prime-boost strategy is an important factor to consider, as with any immunization regimen, and this should be optimized to ensure the most effective response [16]. Currently, few trials are assessing prime-boost strategies as many trials are studying if neoantigen-based vaccines are safe, tolerable, and induce a strong enough immune response.

## 5. Sample Collection and Associated Problems

The technical aspects associated with the identification of personalized neoantigens are immense. To begin the production of a personalized neoantigen, it requires that a sample from the patient must be collected. This sample collection may yield results that indicate this is not an appropriate therapy for the patient. The production process requires a high volume of the tumor sample, that the tumor contains a high number of neoantigens, and has a high mutational burden [19].

The rationale behind the necessity for a high number of neoantigens and a high mutational burden is as follows. The increased number of neoantigens allows the tumor to be recognized and infiltrated by cytotoxic CD8+ T cells. Tumors with fewer numbers of neoantigens may be a target for other immunotherapy or therapeutic vaccinations but represent a problem for the neoantigen-based vaccine due to the restricted number of immunogenic epitopes present on the tumor. A reduction in neoantigens has been identified with metastatic pancreatic cancer after primary resection, indicating the tumor cells have immunoedited and escaped detection from the immune system [16].

Though this rationale makes sense for a number of neoantigens predicting a positive or negative response to immunotherapy, some types of cancer like melanoma and renal cell carcinoma defy this pattern. Mutations present in melanoma and renal cell carcinoma are lower in quantity as compared to other cancers, but the “quality” of the neoantigen may be higher. This concept of a quality neoantigen is based on the fact that renal cell carcinoma has a high level of the insertion and deletion types of mutation, which continuously shift the open reading frame. Further research is necessary to understand how both the quality and quantity of the neoantigen affect patient outcomes [19].

## 6. Production Cost and Time

A significant limitation to personalized vaccines is the cost and time of production. Personalized immunizations require initial gene sequencing as well as validation and production, and this remains an extremely expensive process. Zhang et al. stated that this cost is the biggest challenge behind this therapy [1].

Different vaccine platforms such as peptides, RNA, DNA, dendritic cells, and viral vectors influence the cost of therapy. This choice will influence how quickly a vaccine can be generated [16]. As vaccines are generated under good manufacturing practices and in large quantities, scaling up the production of small nucleic acids and associated delivery technology remains a challenge [1]. The cold-chain requirements necessary to prevent nucleic acid degradation also pose a challenge [4]. While a peptide vaccine would likely be the most cost effective option, an mRNA vaccine would be the most time efficient. The timing of immunization is a particularly important consideration given the fact that most patients are being treated for late stages of cancer and may not survive neoantigen production [1,3].

In order to better assess the challenges behind production and feasibility, one company, Gritstone Oncology, Inc., completed a clinical trial (NCT03794128) with the goal of developing two different neoantigen-based vaccines. The first vaccine was based on next-generation sequencing of the patient’s blood and tumor specimens and prediction of the neoantigens. The second vaccine was generated as a generic vaccine based on patients who shared tumor neoantigens and compatible HLA alleles. As this trial was a manufacturing test run, participants had no intervention. No results have been published for this trial [25].

## 7. Adjuvants

An important consideration in vaccine design is the choice of adjuvants, which are compounds that strengthen the immune responses against the antigens introduced. Adjuvants have also been proven to boost the biological half-life of vaccines, increase antigen uptake by APC, induce local inflammation, and promote cellular recruitment [26].

In earlier studies, most often the adjuvant of choice was poly-ICLC since it is compatible with peptide, mRNA, dendritic cells, and DNA formulations. Poly-ICLC is classified as a synthetic double stranded-RNA mimic and induces an innate immune response via TLR3 and MDA5 pattern recognition receptors. Neimi et al. found that poly ICLC was utilized in 30% of neoantigen clinical trials. The next most common choice was GM-CSF, found in 7% of these trials. It is important to note that approximately half of the neoantigen-based vaccine trials did not use an adjuvant [3].

Though the traditional definition of adjuvants is agents added to non-specifically increase the immune response, one loophole to this definition is dendritic cells, or “nature’s adjuvants [27]”. Zhang et al. published a comparison of a neoantigen adjuvant and neoantigen-pulsed dendritic cell in a murine lung carcinoma model. The adjuvants chosen for these studies were Freund’s adjuvant and incomplete Freund’s adjuvant as compared to the dendritic cell vaccine. The results illustrated the neoantigen-pulsed dendritic cell vaccine has a superior response [26].

More work is needed to determine the best possible adjuvant or potentially utilize a dendritic cell immunization model.

## 8. Comparison of mRNA vs. Peptide Vaccines

mRNA- and peptide-based cancer vaccines constitute the major forms of design to produce vaccines useful for cancer immunotherapies [28,29], and several clinical trials have been conducted with such formulations [30,31,32]. These clinical trials are classified as mRNA- or peptide-based vaccinations. mRNA and peptide vaccines both pose many advantages and disadvantages, besides production and cost which were previously discussed in Section 6.

Given the successful generation of two mRNA vaccines in response to the SARS-CoV2 pandemic, interest in an application of this type of vaccine to cancer has increased. There are several advantages to this type of design, with two types studied for cancer immunotherapy: ex vivo mRNA-loaded dendritic cell vaccines and mRNA-lipid nanoparticle vaccines. Both lead to the same immune response, in which the mRNA encoding for a TAA or TSA provokes an antitumor reaction via CD 8+ T lymphocytes activity. However, the dendritic cell vaccine approach requires the isolation, cell culture, and differentiation of monocytes or hematopoietic progenitor cells into dendritic cells. Though this process is more involved, it has been shown to induce an adaptive immune response and is a safe type of vaccine, with rare adverse events reported [33]. The mRNA-lipid nanoparticle approach directly builds off the foundation established by the technology from the SARS-CoV2 pandemic [34] and has also been shown to induce favorable results in patients with solid tumors and is safe. Naked mRNA has a low bioavailability of antigens due to a limited mRNA update, and the encapsulation with lipid nanoparticles acts to increase the bioavailability [33].

Peptide-based vaccines pose many advantages and disadvantages. The simplicity, safety, low cost, and extensive studies are all major advantages [26]. The most significant disadvantages are the low immunogenicity, easy degradation, short half life, and potential for the tumor cell to evade detection via mutation [4]. The issue with immunogenicity can be overcome through the addition of an adjuvant to improve the immune response, prevent degradation, and lengthen the half life. More discussion of adjuvants can be found in Section 7.

## 9. Summary of Clinical Trials of Neoantigen-Based Vaccines

A subset of the clinical trials, including GRANITE individualized neoantigen vaccine, “Off the shelf” Mutant-KRAS neoantigen vaccine, “Off the shelf” Nous-209, Nous-PEV, NEO-PV-01, NeoVax, an mRNA-based vaccine, GEN-009, and RO7198457 are summarized below in Table 1.

### 9.1. mRNA-Based GRANITE Individualized Neoantigen Vaccines

In a phase 1/2 clinical trial, NCT03639714, Gritstone Oncology, Inc. tested a modified chimpanzee adenovirus and self-amplifying mRNA in lipid nanoparticles using a prime/boost strategy [35,46]. Using Gritstone’s EDGE model, the patient-specific neoantigens were predicted and inserted into a vector with self-amplifying mRNA [36]. The self-amplifying mRNA is derived from a positive ssRNA alphavirus with the self assembly genes [33]. The vaccine design, prime/boost strategy, and unique self-amplifying mRNA component are strong advantages of this trial.

The study was completed with 29 participants with non-small-cell lung cancer, colorectal cancer with microsatellite stability, or gastroesophageal adenocarcinoma and fell short of the projected 214 participants desired [46]. These patients also received nivolumab and ipilimumab in conjunction with the prime boost immunization. Overall, the vaccine was well tolerated, with four serious treatment-related adverse events (one count of duodenitis, increased transaminases, hyperthyroidism, and pyrexia). Since it was well tolerated, the secondary endpoint assessed immunogenicity, feasibility of manufacturing, and overall survival. Results for this found that participants had long-lasting neoantigen-specific CD8 T cell responses. Of the seven patients with microsatellite-stable colorectal cancer, three had prolonged overall survival. From the analysis of biomarkers of these patients, there was a significant reduction in circulating DNA levels and those with prolonged overall survival [8].

Palmer et al. also theorized the effects of the checkpoint inhibitor therapy. In a smaller subset of the participants, those who received ipilimumab are likely have a broader and more robust immune response to neoantigens; given the small cohort of patients, this result remains challenging to discern [8].

To build upon the results of this phase 1/2 trial, a new phase 2/3 study is underway with an estimated enrollment of 700 participants and is estimated to be completed in March 2027. This trial specifically targets patients with metastatic colorectal cancer because a previous trial demonstrated a 44% molecular response rate (greater than or equal to 50% decrease in ctDNA relative to baseline) in 4/9 patients with metastatic colorectal cancer and had an improved overall survival rate for this subset of patients [47,48].

### 9.2. mRNA-Based “Off the Shelf” Mutant KRAS-Neoantigen Vaccine

In a similar clinical trial, NCT03953235, Gritstone Oncology, Inc. tested this vaccine construct with shared neoantigens in participants with non-small-cell lung cancer, colorectal cancer with microsatellite stability, or pancreatic ductal adenocarcinoma [35,49]. The shared neoantigens are from common driver mutations and include several from the KRAS gene. The advantage of this trial was the fact that shared neoantigens were utilized, meaning this may be produced with ease and no time constraints and applied to multiple different cancer types. This also meant that the immunization was not personalized, which may be considered an advantage or disadvantage.

Preliminary data show that one of three patients had a significant KRAS G12C-specific CD8+ T cell response. One patient in this trial had a twenty percent reduction in tumor dimensions associated with a decrease in ctDNA [35,36]. Based on the preliminary results, the personalized neoantigen trial in NCT03639714 has stronger results than this “off the shelf”.

### 9.3. mRNA-Based Therapeutic Vaccine

In a phase 1/2 clinical trial (NCT03480152), Cafri et al. tested an mRNA vaccine in patients with metastatic gastrointestinal cancer [42]. This mRNA vaccine was composed of personalized neoantigens, mutations in expressed driver genes, and HLA-I-predicted immunogenic mutations. The personalized neoantigens were identified through the following protocol. Cafri et al. utilized sequencing data to identify the mutations in metastatic tumor samples. These mutations were generated into long peptides and tandem minigenes. The peptides with mutations combined with autologous tumor-infiltrating lymphocytes were analyzed with high-throughput immunologic sequencing and classified as neoantigens [42]. The advantage of this trial was the fact that a different cancer target was used and that the investigators used a different computational model than Gritstone Oncology’s EDGE model.

Though the initial clinical trial proposal included a phase 2 component, there was no clinical response observed so it was terminated. This meant that there was no increase in the frequency of T cells specific for the neoantigens or tumor shrinkage observed. The lack of clinical response may be explained by previous treatment protocols that depleted tumor-infiltrating lymphocytes, low immunogenicity associated with mRNA vaccination, or other clinical or physical aspects [42].

Though this trial did not improve patient outcomes, it provided important information to the scientific community. The results indicate that this type of vaccination was safe and could potentially be used in adjunct with other therapies. It also gave a benchmark for the time of sample processing and manufacturing because these vaccines were generated within 42 to 60 days [42]. Other researchers estimated the time to identify neoantigens and generate the vaccine as 3–5 months [19].

### 9.4. Peptide-Based NeoVax

NeoVax is an important immunization tested in several phase 1/1b clinical trials and current ongoing trials with additional cancer types. The NeoVax vaccines contain up to 20 different synthetic long peptides (15 to 30-mers) and most commonly are combined with Poly-ICLC (Hiltonol), a proinflammatory agonist for TLR3 and MDA5, as previously discussed in Section 7 [35]. Based on evidence from the previous trial (NCT01970358), NeoVax induced CD4+ T cell and CD8+ T cell responses and could be combined with immune checkpoint inhibitors, which is discussed in more detail in the following paragraph. It also showed that it could produce such responses in immunological cold tumors with low mutational burdens such as MGMT promoter unmethylated glioblastoma [16].

Previously, NeoVax was administered to patients with surgically resected stage IIIB/C or Iva/b melanoma in phase 1 clinical trial NCT01970358 and yielded positive outcomes, with six of eight patients without signs of active disease. Hu et al. tracked the long-term effects of NeoVax and identified persistent vaccine-induced neoantigen-specific T cell responses in all eight patients. This response was mostly sustained four and a half years after vaccination, suggesting that memory T cells were generated. Though some patients had a complete response to treatment, five of eight patients reported melanoma recurrence, which suggests that vaccination alone may not yield long-term antitumor immunity [39].

Several phase 1 trials (NCT02950766, NCT04024878, NCT03929029, NCT03219450, and NCT02287428) are in progress assessing NeoVax’s safety and immunogenicity in different cancer types including renal cell carcinoma [50], ovarian cancer [51], chronic lymphoblastic leukemia [52], melanoma [53], and glioblastoma [54].

To test NeoVax’s potential role in conjunction with other therapies, there are three separate trials to discuss, one with melanoma and two with glioblastoma. For melanoma, there is an ongoing phase 1b clinical trial, NCT03929029, studying melanoma where participants receive NeoVax with Monatanide and checkpoint inhibitors (ipilimumab and nivolumab) [53]. The administration of NeoVax has been altered to include Montanide, an incomplete Freund’s adjuvant [55], or water-in-oil emulsion [3], which has been added to provide a slow release of the peptides at the injection site [53]. This addition of Montanide also helps improve the cytotoxic T cell responses [3]. One trial (NCT03422094) with NeoVax administered in conjunction with ipilimumab and nivolumab for glioblastoma was terminated due to the manufacturer [38]. Currently, a different trial (NCT02287428) investigating NeoVax in glioblastoma is ongoing. Participants in this trial potentially receive combinations of radiation, pembrolizumab, and temozolomide. The rationale behind using temozolomide is dependent on if the patient’s glioblastoma is positive for methylguanine methyltranserase (MGMT), a DNA repair protein that fixes the tumor cell DNA. Only patients who have glioblastoma positive for MGMT will receive this chemotherapy drug. This may yield more effective treatment of this cancer [54].

### 9.5. Peptide-Based GEN-009

Researchers investigated a personalized vaccine composed of 4–20 synthetic long peptides and poly-ICLC in a phase 1/2 clinical trial (NCT03633110). The neoantigens were determined by ATLAS, a bioassay screening method [16,56]. This screening method is said to “screen each patient’s mutanome to identify neoantigens for vaccine inclusion and deleterious inhibigens (trademark)for exclusion” without predictions [44]. The vaccine regimen was combined with anti-PD-1 checkpoint inhibitor therapy. The advantage of this trial was the peptide basis of vaccine and method of discovery of neoantigens.

In the preliminary results, it was found to be a safe and tolerable vaccine, with all participants having both CD4+ and CD8+ T cells responses to neoantigens. The vaccination increased the IFN gamma levels, and this response was found for at least 6 months in some patients [43]. Interestingly, the majority of the T cell responses lasted for more than 12 months [16] and some patients even had no circulating tumor DNA, according to the preliminary data presented by Gillison and Shainheit [43,44]. It was also found that neoantigen-specific responses had an association between the quantity and kinetics of cytokine section and increased Ki-67+ T cells and TEM cells [44]. More data are necessary regarding the overall results and details of patient outcomes.

## 10. Emerging Targets in Cancer Immunotherapy

The established immunotherapies that target surface receptors such as CTLA-4 and/or PD-1 with recombinant antibodies have been crucial for cancer treatment, but a significant population of patients fail to respond to these immunotherapies and a low number of them are finally cured [57]. The recent progress suggests that, in addition to surface receptors, intracellular proteins could also be the target of cancer immunotherapies [58]. One of the such targets, the nuclear receptor NR2F6 (nuclear receptor subfamily 2 group F member 6, also called Ear-2), has been shown to be an intracellular immune checkpoint in effector T cells. The targeting of NR2F6 for cancer immunotherapy has the potential to increase the response rates of cancer patients and/or to extend treatment to a broader range of cancer types [59].

Furthermore, in addition to neoantigens, newer strategies are being explored that target additional immunomodulatory pathways to activate the patient’s own anti-tumor immune responses. These emerging targets belong to co-inhibitory and co-stimulatory molecules of the innate and adaptive immune system and include: 1. T lymphocyte markers: Lymphocyte Activation Gene 3 [LAG-3], T cell ImmunoGlobulin and ITIM domain [TIGIT], T cell Immunoglobulin- and Mucin-domain-containing molecule 3 [TIM-3], V-domain containing Ig Suppressor of T cell Activation [VISTA], B7-H3, Inducible T cell Co-stimulator [ICOS/ICOS-L], CD27/CD70, and Glucocorticoid-Induced TNF Receptor [GITR]; 2. natural killer cell markers: CD94/NKG2A and the Killer Immunoglobulin-like receptor [KIR] family; and 3. macrophage markers: CD47/Signal-Regulatory Protein alpha [SIRPα], and Indoleamine-2,3-Dioxygenase [IDO], etc. [60]. These emerging immune targets have shown pre-clinical efficacy with progression to active investigation in clinical trials [60,61]. Several therapeutic mRNA-based cancer vaccines as a monotherapy are undergoing clinical trials [62]. Although therapeutic mRNA-based cancer vaccines are yet to be approved as a standard treatment, encouraging results are being reported from clinical trials [63].

## 11. Future Directions

Multiple avenues must be explored to produce a safe and effective therapeutic cancer vaccine. These avenues begin with target discovery from the tumor biopsy, then vaccine formulation and administration, and finally characterization of immune response. As previously discussed, determination of the neoantigens from the sample involve in silico algorithms and require neoepitope validation. The next avenue of optimization is the vaccine formulation, administration, and regimen. This includes the type of vaccine, neoantigens selected, sequencing and dosing of the vaccine, and the combination of other therapeutics selected. The final stage is to assess how the vaccine works and the associated phenotypes of CD4+ and CD8+ T cells [16]. Given how broad these three categories are, different emerging technologies likely will play a role in the final cancer vaccine.

Emerging technology impacting the first stage of target discovery is primarily advances in biotechnology and bioinformatics. Data mining and next-generation sequencing, whole exome sequencing, and their associated analyses all fall under this category. [19]. Additionally, “clustered Regularly Interspaced Short Palindromic Repeats” or CRISPR/Cas9 could potentially be applied to identify the tumor gene and permit researchers to edit the gene of interest. This strategy would override any issues with tumor heterogeneity [64].

In terms of emerging technologies that show promise for the formulation stage, alternative delivery techniques and nanovaccine technology are important concepts to consider. The alternative delivery techniques explore options outside of the more conventional vaccines and drug formulations. These range from in vivo nanoparticle delivery to immune cells, ex vivo T cell functionalization with nanoparticles, biomaterial implant scaffolds, injectable biomaterial scaffolds, and the transdermal delivery method, as reviewed by Rachel Riley in “Delivery technology for cancer immunotherapy [15]”. Any of these delivery methods may yield efficient and cost-effective treatment options. Exploring nanovaccine technology also opens the field to adjuvants and biomaterials like chitosan, collagen, hydraluron, synthetic polymers, lipid nanocarriers, or inorganic nanomaterials as discussed by Shengxian Li et al. However, more research is needed to ensure the safety and immunogenicity [65]. Though these concepts both show promise, there are still multiple avenues already being explored, as evidenced by the clinical trials. It is hopeful that building off of the knowledge of these trials and exploration into these delivery methods will result in a therapeutic vaccine.

One component to vaccine regimen is the other therapeutics selected, like chemotherapy, radiation, or immunotherapy options. The immunomodulating antibody options are expanding as more drug targets are discovered and studied. Though PD-1/PD-L1 and CTLA-4 checkpoint inhibitors drastically advanced the field of immunotherapy and expanded treatment options, there are still challenges with these inhibitors. More work into studying other pathways like DNA damage repair, anti-angiogenic targets, and alternative checkpoint inhibitors may yield more effective treatments or personalized treatment options [7].

Future directions for the final stage of immune assessment is a critical component to this process. This immune assessment identifies the effect of the vaccine based on the phenotypes of the CD4+ and CD8+ T lymphocytes and the correlates of tumor control improvement. This will likely involve high-resolution immunophenotyping with single-cell RNA-seq and a single-cell T Cell Receptor analysis [16].

## 12. Conclusions

The developments in cancer therapeutic neoantigen-based vaccines are promising and progress in tandem with improvements in neoantigen identification and manufacturing. There are several challenges to overcome with every stage of development, as reviewed in detail, including neoantigen identification, formulation with regimen, delivery technology, immune validation, and manufacturing issues. With new vaccine designs combined with adjunctive checkpoint inhibitors, multiple clinical trials, displayed in Table 1, illustrate the potential of this multifaceted approach. mRNA-based vaccines like GRANITE neoantigen and peptide-based vaccines like NeoVax and GEN-009 have recently yielded positive outcomes but still need further investigation. Both the both basis behind both vaccines was discussed, with multiple advantages and disadvantages. Also, other immunotherapies like CAR T cell therapy, radiation, or chemotherapy drugs could also be employed to boost the action of an immunization to kill tumor cells. It is important to note that potent adjuvants, alternative delivery methods, and emerging technologies like CRIPR/Cas9 are important and all may play a role in the future of cancer therapeutic vaccines.

## Figures and Tables

**Table 1:** Anti-Cancer Vaccines in Clinical Trials.

Vaccine Type	Summary of Vaccine Formulation	Participants Also Treated with	Type of Cancer Treated	Clinical Trial Phase	NCT Number	Results Summary, If Available
GRANITE individualized neoantigen vaccines	GRT-C901 (vaccine prime) and GRT-C902 (vaccine boost) are chimpanzee adenovirus with self-amplifying mRNA	Nivolumab (anti-PD-1 monoclonal antibody), ipilimumab (anti-CTLA-4 monoclonal antibody)	Non-small-cell lung cancer, colorectal cancer, gastroesophageal adenocarcinoma, urothelial carcinoma	1/2, 2 completed	NCT03639714	Safe, tolerable, and potent immunogenicity. Few serious treatment-related adverse events. Decrease in ctDNA and extended participants’ lives [8]
GRANITE individualized neoantigen vaccines	GRT-C901/GRT-R902	Chemotherapy per standard of care, Ipilimumab (anti-CTLA-4 monoclonal antibody), Atezolizumab (anti-PD-ligand 1)	Metastatic colorectal cancer	2/3, active not recruiting	NCT05141721	NA
“Off the shelf” mutant KRAS neoantigen vaccine prime and vaccine boost	GRT-C903 (vaccine prime) and GRT-C904 (vaccine boost)	Nivolumab (anti-PD-1 monoclonal antibody), ipilimumab (anti-CTLA-4 monoclonal antibody)	Non-small-cell lung cancer, colorectal cancer, pancreatic cancer, solid tumor, shared neoantigen-positive solid tumors	1/2, 2, active not recruiting	NCT03953235	Preliminary results indicate safe and tolerable. One of three patients had neoantigen-CD8 T cell response. [35,36]
“Off the Shelf” Nous-209	GAd20-209-FSP (vaccine prime) and MVA-209-FSP (vaccine boost)	Pembrolizumab (anti-PD-1 antibody)	Microsatellite unstable solid tumors	1/2, 2, currently ongoing	NCT04041310	Preliminary results for phase 1 indicate safe and tolerable. Overall response rate on RECIST1.1 was 67% [37]
Nous-PEV	Personalized vaccine (PEV) based on Gad-PEV (vaccine prime) and MVA-PEV (vaccine boost)	Pembrolizumab (anti-PD-1 antibody)	Non-small-cell lung carcinoma, melanoma	1b, currently ongoing	NCT04990479	NA
NeoVax	Combination of neoantigen peptides and poly-ICLC (Hiltonol)	Ipilimumab	Renal Cell Carcinoma	1, recruiting	NCT02950766	NA
NeoVax	Combination of up to 20 neoantigen peptides and poly-ICLC (Hiltonol)	Nivolumab	Ovarian cancer	1, currently ongoing	NCT04024878	NA
NeoVax plus Montanide	Combination of neoantigen peptides and poly-ICLC (Hiltonol)	Ipilimumab (locally administered) and Nivolumab (systemic)	Advanced melanoma	1b, currently ongoing	NCT03929029	NA
NeoVax	Combination of up to 20 neoantigen peptides and poly-ICLC (Hiltonol)	Cyclophosphamide (chemotherapy drug) and Pembrolizumab	Chronic lymphocytic leukemia	1, currently ongoing	NCT03219450	NA
NeoVax	Combination of neoantigen peptides and poly-ICLC (Hiltonol)	Radiation, Temozolomide (depending on methylation of DNA repair protein), and Pembrolizumab	Glioblastoma	1, currently ongoing	NCT02287428	NA
NeoVax	Combination of neoantigen peptides and poly-ICLC (Hiltonol)	Nivolumab, Ipililumumab	Unmethylated glioblastoma	1, terminated	NCT03422094	Terminated due to the manufacturer switching to cell therapy [38]
NeoVax	Combination of up to 20 neoantigen peptides and poly-ICLC (Hiltonol)	NA	Melanoma (surgically resected stage IIIB/C or Iva/b)	1, completed	NCT01970358	6 of 8 patients had no evidence of active disease. Neoantigen-specific T cell response persisted with evidence [39]
NEO-PV-01	Personalized cancer vaccine NEO-PV-01 with Poly-ICLC (Hiltonol), an investigational adjuvant	Nivolumab (Opdivo trade mark name)	Metastatic or advanced melanoma, lung, or bladder cancer	1b, completed	NCT02897765	Safe, immunogenic response [40]
NEO-PV-01	Personalized cancer vaccine NEO-PV-01 with Poly-ICLC (Hiltonol), an investigational adjuvant	Pembrolizumab, Chemotherapy	Non-small-cell lung cancer, lung cancer, nonsquamous non-small-cell neoplasm of lung	1, completed	NCT03380871	Safe, tolerable, induced immune response, as presented at American Cancer Association Research but further detail needed [41]
mRNA-based personalized cancer vaccine targeting neoantigens	Validated defined neoantigens, predicted neoepitopes and mutations in driver genes into a single mRNA concatemer (mRNA-4650)	NA	Melanoma, colon cancer, gastrointestinal cancer, genitourinary cancer, hepatocellular cancer	1/2, terminated (slow accrual)	NCT03480152	Safe, induced mutation-specific T cell responses against predicted neoantigens, but no tumor shrinkage noted with this trial–likely needs to be utilized with additional therapeutic agent [42]
GEN-009	Synthetic long peptides identified as personalized neoantigens, Poly-ICLC adjuvant	Nivolumab, Pembrolizumab	Cutaneous melanoma, non-small-cell lung cancer, squamous cell carcinoma of the head and neck, urothelial carcinoma, renal cell carcinoma	1/2, 2, completed	NCT03633110	GEN-009 safe, tolerable and found to produce vaccine-stimulated T cell response for more than 12 months. More data for dual therapy are expected [43,44]
RO7198457	Neoantigens in RNA-Lipoplex Neoantigen Specific immunotherapy (iNeST)	Atezolizumab	Melanoma, non-small-cell lung cancer, bladder cancer, colorectal cancer, triple-negative breast cancer, renal cancer, head and neck cancer	1, active not recruiting	NCT03289962	Safe, tolerable. Preliminary data indicate infiltration of RO7198457 stimulated T cells [45]
RO7198457	Neoantigens in RNA-Lipoplex Neoantigen Specific immunotherapy (iNeST)	Pembrolizumab	Advanced melanoma (no prior treatment)	2, active not recruiting	NCT03815058	NA

## Data Availability

Not applicable.
